# Surgical considerations in pediatric necrotizing fasciitis

**DOI:** 10.4103/0971-9261.54816

**Published:** 2009

**Authors:** A. Pandey, A. N. Gangopadhyay, S. P. Sharma, V. Kumar, S. C. Gopal, D. K. Gupta

**Affiliations:** Department of Pediatric Surgery, Institute of Medical Sciences, Banaras Hindu University, Varanasi - 221 005, U.P. India

**Keywords:** Children, debridement, necrotizing fasciitis

## Abstract

**Background:**

Necrotizing fasciitis (NF) is a serious infection of soft tissues. This paper presents experience with pediatric NF and suitability of conservative surgery in its management.

**Materials and Methods:**

In this retrospective study, 70 patients of NF were managed during the study period of eight years. The study was divided into two time periods- first period (June 1998 to June 2001- group 1) and second period (June 2001 to June 2006- group 2). The parameters studied were age, sex, site of involvement and treatment. The treatment included intravenous antibiotics, supportive therapy and either aggressive (group 1) or conservative surgery (group 2).

**Results:**

Age of presentation ranged from 10 days to 11 years. Male to female ratio was 1.69:1. Back was the commonest site to be involved. Culture reports were polymicrobial in 70% with predominance of *Staphylococcus* species. Predisposing factors included malnourishment, boils, scratch injury, intravenous cannulation and injections. Conservative surgery had better outcome in terms of hospital stay, complications and cost of treatment.

**Conclusion:**

NF is a serious and disease which requires immediate and all out attention. Early diagnosis, aggressive supportive treatment and conservative surgery improve survival.

## INTRODUCTION

Necrotizing fasciitis (NF) is a life threatening condition consisting of soft tissue infection with rapid progression and wide spread fascial necrosis.[1] All age groups, including neonates can be affected.

The mainstay of treatment is antibiotics and surgery which consists of debridement. In the standard management of NF, both for children and adults early and aggressive debridement is advocated.[1–5] There are some studies suggesting that aggressive surgery can be delayed until the patient settles which may help decrease morbidity of the condition.[6–8] Hence, an important point of consideration is timing and type of surgery in cases of NF. This study was done to see the trends of NF along with its surgical considerations and efficacy of treatment with delayed surgery. The purpose of the study was to see if delayed debridement benefits children and neonates.

## MATERIALS AND METHODS

This was a retrospective study done from June 1998 to June 2006. All NF patients admitted were studied. The diagnosis of NF was made on clinical and microbiological basis. Patients were studied for age, sex, area of involvement, microbiology, duration of stay, and predisposing factors.

The study was divided in to two time periods. In the first period (June 1998 to June 2001), early aggressive surgery was done (group 1). During this period, debridement was done immediately after the diagnosis of NF was made either in the cellulitic stage, with dusky blue patch or when gangrene of the involved skin had occurred. It involved complete removal of the necrotic tissue until the viable bleeding tissue was visible under the necrotic bed. The procedure was carried out in the operation theatre under general anesthesia. Antibiotics were used to cover gram positive (Ceftriaxone 50 mg/kg IV 12 hourly or Co amoxycalv 30 mg/kg IV 8 hourly), gram negative (Amikacin 7.5mg/kg IV 12 hourly) and anaerobes (Ornidazole 10 mg/kg IV 12 hourly). IV fluid was given to replace volume deficit. Analgesia was provided by intramuscular (IM) Pentazocine (0.5mg/kg). If required, IM Diazepam (0.1to 0.3 mg/kg) was given for restlessness and agitation.

The second time period ranged from June 2001 to June 2006 (group 2). The management included blood investigation (Hemogram, renal function tests) before and after treatment, blood transfusion, intravenous (IV) antibiotics and debridement. The antibiotics and supportive management were same as for the group 1. In both the groups, the counts were repeated on alternate days.

**Figure 1:**
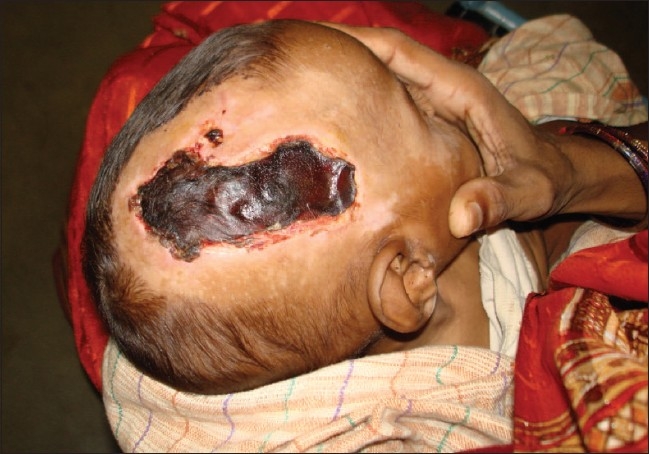
Necrotic black patch separated from the surrounding healthy skin in a three-month-old child four days after admission and conservative management

**Figure 2:**
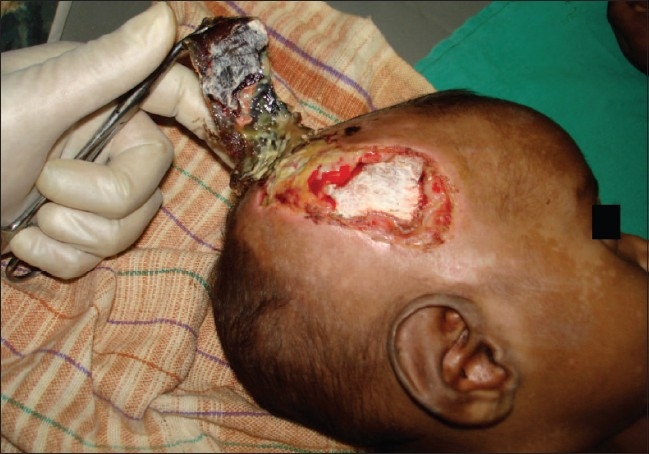
Careful removal of the patch with minimal blood loss

**Figure 3:**
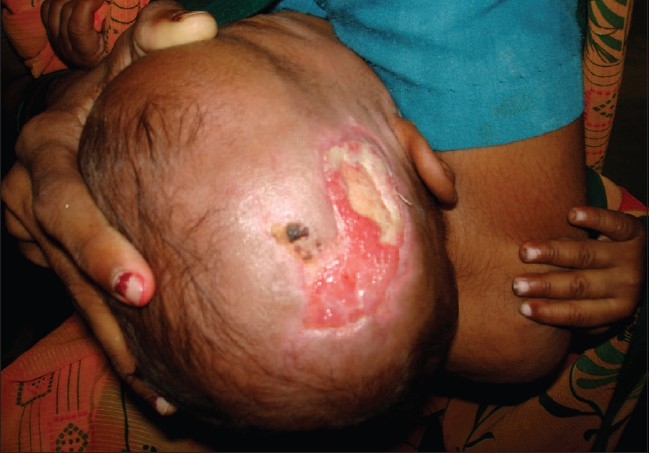
Healthy granulation tissue visible over the wound area after 17 days of treatment

In group 2, debridement was done three to five days after starting antibiotics intravenously and on formation of dry black skin loosely adhered to its base and separating from surrounding healthy skin [Figure 1]. This procedure was done under sedation. It included careful removal of only dead skin [Figure 2] along with daily washing with normal saline and dressing with povidone iodine solution and placentrex gel. No surgical intervention was attempted in acute stage. Dressing was done repeatedly with povidone iodine lotion and placentrex gel (Human placental extract, Albert David, Kolkata, India), both before and after debridement till the wound had healthy granulation tissue [Figure 3]. The patients were discharged when they were afebrile, taking orally, blood counts were normal (white blood cells WBC less than 11000/mm^3^) and the wound was healthy and granulating.

The cost of treatment was evaluated on the basis of all expenses needed for surgery, medications, investigations and blood transfusions. This also included the expenses incurred by the hospital on the patient such as free drugs etc. The outcome was evaluated on the basis of hospital stay, complication and mortality.

All the data was entered into the Microsoft Excel sheet. The statistical analysis was done using SPSS 12.0 version for Windows. The analysis was done using paired *t-test*, Chi square test, Fisher exact test and one way ANOVA. The values are expressed as mean plus/minus standard deviation. The *P* value of less than 0.05 was considered as statistically significant.

## RESULTS

The total number of patients in groups 2 and 1 was 49 and 21 respectively. Both the groups had comparable age and sex distribution [Table 1]. In both groups, the mode of presentation was cellulitis followed by a rapidly developing black patch of skin that was preceded by features of septicemia. In group 2, these included fever 43 (87.7%), lethargy 41(83.6%), anorexia 40 (81.6%), seizures two (4%) and loss of consciousness one (2%). In group 1, the features of septicemia noticed were fever 18 (85.7%), lethargy 18 (85.7%) and anorexia 15 (71.4%). Thus, both the groups had comparable mode of presentations (*P* greater than 0.05).

**Table 1:** Comparative evaluation: Conservative surgery, aggressive surgery groups

	Conservative surgery group 2 (n = 49)	Aggressive surgery group1 (n = 21)	*P* value
Age	3.54 ± 3.87 years (range 10 days to 11 years)	3.35 ± 3.26 years (range 10 days to 10 years)	>.05
Male: female	1.72:1	1.63:1	>.05
Initiating agent	42 (85.7%)	19 (90.48%)	>.05
Culture reports	Polymicrobial in 32 (65.3%)	Polymicrobial in 16 (76.19%)	>.05
Normal blood count	6.14 ±.76 days	8.57 ±.98 days	<.001
Hospital stay	15.29 ± 2.33 days	22.29 ± 4.33 days	<.001
No. of blood transfusions	1.89 ± 0.77	3.24 ± 0.70	<.001
Appearance of granulation tissue^1^	8.55 ± 0.91 days	13.29 ± 1.35 days	<.001
Mortality	10 (20.4%)	5 (23.81%)	>.05

1 -This meant presence of a layer of granulation tissue over the wound bed

In group 2 the area of involvement included back 18 (36.7%), neck and scalp nine (18.3%), thigh eight (16.3%), abdomen six (12.2%), chest five (10.2%) and arm three (6.1%). In group 1 the area of involvement included back 10 (47.62%), thigh four (19.05%), neck and scalp three (14.29%), abdomen two (9.52%) and arm two (9.52%). Both groups were comparable for the area of involvement [*P* greater than 0.05, Table 1].

In group 2, the culture reports were polymicrobial in 32 (65.3%) and rests were unimicrobial. There was predominance of *Staphylococcus* species (73.4% of total), *S. aureus* being 30 (61.2%) and *S.epidermidis* six (12.2%), others being Streptococcus six (12.2%), *E. coli* four (8.1%) and *Klebsiella* three (6.1%). In group1 polymicrobial culture reports were noticed in 16 (76.19%) patients, rest being unimicrobial. Both the groups were comparable regarding the type of culture [*P* greater than. 05, Table 1]. *Staphylococcus* species predominated in this group also (80.95% of total).*S. aureus* was noticed in 13 (61.9%), *S. epidermidis* 4 (19.05%), Streptococcus three (14.29%) and *E. coli* one (4.76%).

Hemogram revealed hemoglobin in range of seven gm % to 12 gm % (mean 8.23 plus/minus 1.39 gm %) in both the groups. The serum urea and creatine levels ranged from 15 to 39 mg % (mean 21.23 plus/minus 6.17 mg %) and 0.5 to 1.1 mg % (mean 0.66 plus/minus 0.15 mg %) respectively in both the groups. Total leukocyte counts were above 13000/mm^3^ with predominance of neutrophils (greater than 75%). C reactive protein (CRP) was done in 21 patients in group 2 and was uniformly raised. It was not done in rest of patients due to cost factor. In group1 CRP was not done in any of the patients because of unavailability of the facility at that time. Blood transfusion raised the hemoglobin to the level of 12 gm % to 15 gm % (mean 13.5 plus/minus 1.06 gm %) in both the groups. Total and differential leukocyte counts (TLC and DLC) normalized after five to seven days (mean 6.14 plus/minus.76 days) of treatment in group 2 and seven to 10 days (mean 8.57 plus/minus.98) in group 1 [*P* less than 0.001, Table 1]. Intravenous antibiotics were continued for at least 10 days.

Patients were discharged after having healthy granulating wound to be healed by secondary intention or skin grafting. In group 2 36 (73.4%) patients were allowed to heal by secondary intention and the remaining 13 (26.5%) had skin grafting. In group 1 14 (66.67%) patients had healing by secondary intention and seven (33.33%) had skin grafting. The duration of hospital stay was significantly higher in group 1 [Table 1].

In groups 1 and 2 complications occurred in 10 (52.38%) and 11 (20.4%) patients respectively. These included septicemias, secondary wound infection, disseminated intravascular coagulation and functional disability [Table 2].

**Table 2:** Comparative evaluation of two treatment modalities on the basis of type of complication

	Conservative surgery group 2 n (%)	Aggressive surgery group 1 n (%)	*P* value
Sepsis	10 (20.04)	6 (28.57)	0.045
Wound infection	8 (16.33)	9 (42.86)	0.018
DIC	4 (8.16)	3 (14.29)	0.434
Disability	3 (6.12)	3 (14.29)	0.264

The *P* value is significantly higher for the wound infection.

Secondary wound infection was suspected on re appearance of pus on the wound site or fever. The percentages in brackets are as per the number of patients in the respective groups. There can be more than one complication in a patient; hence, number of complications is greater than the total number of patients having the complications

In group 2, 10 patients (20.4%) expired. Three of them were premature neonates. All had poor general condition and didn't respond to the treatment. Predisposing factors included malnourishment 30 (61.2%), boils 29 (59.1%), scratch injury six (12.2%), intravenous cannulation three (6.2%), and injections two (4.1%). In group 1, five (23.81%) patients expired [*P* greater than. 05 for mortality, Table 1].

On comparing this modality with the approach of aggressive early debridement, we found conservative surgery to be superior to conventional surgery [Tables 1–3]. The results were statistically significant in terms of hospital stay, complications and cost of treatment.

**Table 3:** Comparison of the two treatment modalities

	Conservative surgery	Aggressive surgery
Cost of treatment^1^	Rs. 9510.20 ± 1038	Rs. 14071.03 ± 2232
Complications n (%)^2^	11 (22.4%)	10 (47.6%)

It included cost of surgery, medications, investigations and blood transfusions (*P* < 0.001). This also included the expenses that the hospital did for the patient; Chi square value 4.44 (*P* < 0.05). Fisher exact test 0.048. n refers to the number of patients having complications and there can be more than one complication in a patient

## DISCUSSION

NF is seen primarily in adults[2] but there are some pediatric series also.[2–4,8–10] Overall male to female ratio in our study was 1.69:1. Legbo *et al*. reported the ratio to be of 1.7:1,[10] suggesting a slightly higher incidence in male patients.

The commonest area of involvement in our study was back which corresponds with other studies.[2,10] Most of the patients had history of some inciting agent detailed in the results with the predisposing factors such as boils, scratch etc. There is a possibility of some cause in rest of the patients which the attendants probably failed to notice. The common inciting factors reported in literature are minor injuries[11] surgical and traumatic wounds,[12] varicella and immunosuppression.[3] It has been shown to be associated with immunodeficiency and even HIV infection in case reports.[13,14]

Majority of the culture reports were polymicrobial which is in accordance with the various retrospective studies and reviews, predominant species being *Staphylococcus* and *Streptococcus*.[8,10,11,15] Our patients belonged to poor socio-economic class with poor nutritional status and hence poor immunity. NF can be severe in such condition, probably due to improper care given to the children and ignorance in this section of the society. The cost effective method adopted by us was transfusion of fresh blood in every patient we treated. It also helped in building up of hemoglobin which is frequently low in this part of the world. TLC and DLC were prime indicators of response to treatment as normalization of counts occurred after 5 to 10 days of intravenous antibiotics. It can be argued that fresh blood contains leukocytes which can increase the TLC, thereby blunting our assessment that is based on normalization of counts. We, however, believe that it is not possible as neutrophils, which form major pool of leukocytes survive in circulation for about six hours[16] and we measured TLC on every alternate day.

Early diagnosis is important for early start of treatment. There are various reports of newer diagnostic modalities like USG, CT and MRI to help in early diagnosis with good results.[17–19]

Supportive therapy is needed in form of intravenous fluids, pain control[20] and oxygen supplementation if needed. As literature suggests predominance of polymicrobial flora in NF,[21] our reports despite being unimicrobial or polymicrobial we used ceftriaxone/co amoxyclav for gram positive, aminoglycoside for gram negative and ornidazole for anaerobic organisms as first line drugs. On the basis of culture reports and clinical response others drugs were used viz. sulbactam and cefoparazone, cefipime as per their dose with good response.

We adopted what we call as conservative surgery in managing the patient after noticing some success with delayed debridement in the literature.[6,7] We were able to achieve healthy granulating wound in all patients in whom conservative debridement was done. For tissue healing, we used the Placentrex gel which is human placental extract. It promotes healing and is also claimed to be having some anti inflammatory activity. There are reports of conservative management of NF with good results.[6–8] We don't prefer the aggressive surgery for the condition like others.[2–5,9,11]

According to the proponents of aggressive surgery, total excision of all necrotic tissues including muscle, fascia and skin is needed to control the progression of NF and ongoing release of bacterial toxins.[9] In various retrospective studies and reviews, early and aggressive debridement has been recommended for favorable results;[2–5,9–11] however, none of them has commented up on delayed debridement, suggesting that it was never considered as an initial choice. We are in accordance of conservative surgery like Wakhlu *et al*.[8] as we believe that extensive early surgical debridement in the acute stage of infection requires multiple blood transfusions and intensive care management and adds surgical stress over the systemic effects of the infection. It is hypothesized that debridement during the acute stage of NF may promote entry of the infecting organism through newly opened vascular channels leading to flaring of the infection. Though Bingol-Kologlu *et al*.[9] recommended early debridement, it is to be noticed that cutaneous gangrene had already occurred because of delayed admission in their patients which is more or less like delayed surgery.

Our results show that conservative surgery is worth attempting as an initial choice [Tables 1–3]. Though complications were not statistically significant in both the groups except secondary wound infection [Table 1], we believe that it was due to the less number of patients in the group1. Moreover, overall percentage of complications was greater in group1 and was statistically significant [Table 3].

The limitation of our study could be smaller group of patients in which aggressive surgery was done (group 1). This may be a reason for insignificant values for the complications discussed in the Table 2. The other limitation of the study could be historic controls; however, as this was not a randomized control trial we had to rely on them.

To conclude, NF in children can be a serious disease which requires immediate and all out attention. High index of suspicion, early diagnosis, aggressive supportive treatment and conservative surgery instead of aggressive surgery as and when needed may improve survival. A large prospective study comparing both the treatment modalities may throw additional light on the feasibility of conservative surgery.
